# From Glycemia to Grip: A Comprehensive Review of Musculoskeletal Complications in Diabetic Patients

**DOI:** 10.7759/cureus.90838

**Published:** 2025-08-23

**Authors:** Saeed Hussain, Muhammad Irfan, Mamoon Khan, Muhammad Kamran Khan, Saira K Awan, Syed S Raza, Abid Nawaz Khan Adil, Giustino Varrassi

**Affiliations:** 1 Acute Medical Unit, Blackpool Teaching Hospitals UK, Blackpool, GBR; 2 Internal Medicine Training, East Suffolk and North Essex NHS Foundation Trust, Colchester, GBR; 3 Diabetes and Endocrinology, University Hospitals of Derby and Burton, Derby, GBR; 4 Medicine and Surgery, Khyber Medical College, Peshawar, PAK; 5 General Internal Medicine, Blackpool Victoria Hospital, Blackpool, GBR; 6 Neurology, Advanced Neurology P.C., Brooklyn, USA; 7 Medicine, Kabir Medical College, Peshawar, PAK; 8 Family Medicine, The Wright Center for Graduate Medical Education, Scranton, USA; 9 Internal Medicine/Physiology, Khyber Medical College/Khyber Teaching Hospital, Peshawar, PAK; 10 Physiology, Khyber Medical University, Peshawar, PAK; 11 Internal Medicine, California Health Science University, Clovis, USA; 12 Internal Medicine, Community Medical Center, Fresno, USA; 13 Pain Medicine, Fondazione Paolo Procacci, Rome, ITA

**Keywords:** capsulitis, carpal tunnel syndrome, charcot arthropathy, diabetes, hyperglycaemia, musculoskeletal

## Abstract

Diabetes mellitus is a chronic metabolic disorder with well-established vascular complications. Its impact on the musculoskeletal (MSK) system is often underrecognized in clinical practice. MSK disorders contribute significantly to pain, functional limitations, and decreased quality of life in individuals with diabetes. However, they remain inadequately addressed in primary care settings. This narrative review aims to synthesize current evidence on the prevalence, assessment, pathophysiology, clinical features, and management of MSK complications in individuals with diabetes, with an emphasis on the role of primary care providers (PCPs) in early detection and management. A comprehensive literature search was conducted using PubMed, Google Scholar, and Scopus to identify peer-reviewed studies published between 2000 and 2025. Findings were organized thematically. MSK complications, including diabetic cheiroarthropathy, trigger finger, adhesive capsulitis, carpal tunnel syndrome (CTS), diffuse idiopathic skeletal hyperostosis (DISH), and Charcot joint, are significantly more prevalent in diabetic populations compared to non-diabetics. These conditions are driven by hyperglycemia-induced biochemical changes, chronic inflammation, and microvascular dysfunction. Primary care interventions encompass lifestyle modification, physical therapy, and pharmacologic management. These can help mitigate disability and improve patient outcomes. Despite the burden, diabetes-related MSK disorders are often overlooked due to time constraints, lack of awareness, and absence of standardized guidelines. Integrating MSK assessment into routine diabetes care is essential to reduce morbidity and preserve mobility in individuals with diabetes. PCPs play a central role in early identification and management of these conditions. Patient education, multidisciplinary collaboration, and future development of evidence-based screening protocols are critical in improving management of diabetes-related MSK complications.

## Introduction and background

Diabetes mellitus (DM) is a chronic metabolic disorder. It is characterized by hyperglycemia that results from defects in insulin secretion, insulin action, or both [1]. In addition to the well-known macrovascular and microvascular complications of diabetes, it is increasingly recognized for the musculoskeletal (MSK) manifestations. These significantly contribute to the functional impairment and decreased quality of life in affected individuals [2].

The prevalence of MSK disorders is considerably higher among individuals with diabetes. Population-based studies indicate that 35-84% of diabetic patients experience MSK pain or dysfunction. There is nearly twice the rate observed in non-diabetic populations [3,4]. These conditions are often underrecognized in routine diabetes care despite their impact on work productivity, daily functioning, and overall health status [5].

Several pathophysiological mechanisms link diabetes with MSK disorders. Chronic hyperglycemic states lead to the accumulation of advanced glycation end-products (AGEs), which stiffen collagen and other structural proteins, contributing to joint immobility and soft tissue thickening [6]. Furthermore, oxidative stress and systemic inflammation seen in diabetes accelerate degenerative processes in periarticular tissues and joints [7].

Common diabetes-related MSK conditions include adhesive capsulitis, carpal tunnel syndrome (CTS), diabetic cheiroarthropathy (DC), trigger finger, diffuse idiopathic skeletal hyperostosis (DISH), and Charcot arthropathy [2,8,9]. These disorders show a spectrum of severity, ranging from mild discomfort to severe deformity and disability. Timely intervention and early detection can prevent progression and improve patient outcomes.

Primary care providers (PCPs) are not only at the forefront of managing chronic diseases but are also very well positioned to identify MSK complications during routine diabetes care. In resource-limited settings, where access to specialists may be delayed, the role of PCPs is critical. Additionally, comprehensive care by PCPs includes performing focused physical exams, screening for MSK symptoms, and initiating timely referrals or conservative management [10].

Despite the importance, guidelines rarely address MSK complications as part of diabetes care algorithms. This may lead to missed opportunities for early intervention. There is a growing need to integrate MSK assessment into diabetes management protocols and raise awareness among PCPs about these conditions [10]. This review aims to fill that gap by summarizing current knowledge, evidence-based interventions, and diagnostic strategies for MSK pain in diabetic patients within the primary care context.

## Review

Methodology

This narrative review was conducted to synthesize clinical guidance and the current evidence on the assessment and management of MSK complications in diabetic patients. A structured approach was used to identify clinical guidelines, relevant literature, and expert consensus documents. This review was conducted in accordance with the Scale for the Assessment of Narrative Review Articles (SANRA) guidelines to ensure methodological rigor and clarity [11]. The framework provided guidance in planning, literature selection, synthesis, and presentation of the findings related to the MSK complications in diabetic patients. 

Search Strategy

This narrative review was conducted by searching and synthesizing evidence from peer-reviewed articles published between 2000 and 2025. Databases including MEDLINE, PubMed, Google Scholar, EMBASE, Scopus and Cochrane Library were queried using search terms such as “diabetes mellitus,” “musculoskeletal disorders,” “primary care,” “MSK pain,” “cheiroarthropathy,” “adhesive capsulitis,” and “Charcot joint.” Major guidelines were also hand searched from agencies such as American Academy of Family Physicians (AAFP), Centers for Disease Control and Prevention (CDC), and World Health Organization (WHO) to identify additional relevant sources.

Inclusion Criteria

Inclusion criteria comprised English-language studies, systematic reviews, meta-analyses, and clinical guidelines that discussed the prevalence, pathophysiology, diagnosis, or management of MSK disorders in diabetic populations. Additional reference lists from key studies were manually reviewed for relevant citations.

Exclusion Criteria

Non-peer-reviewed articles, articles focusing solely on pediatric populations, surgical interventions or specialist-only care were excluded to maintain relevance to primary care practice. Opinion pieces and studies exclusively written in non-English were also excluded.

Data Extraction and Synthesis

The review identified several key themes essential to understanding and improving the assessment and management of MSK complications in individuals with diabetes. Data extraction and synthesis focused on identifying common MSK conditions associated with diabetes, their epidemiological relevance, diagnostic approaches, and management strategies in primary care. Relevant data were extracted from selected articles based on inclusion criteria and organized thematically. Findings were synthesized to highlight patterns, clinical recommendations, and gaps in current practice, with particular attention to both pharmacologic and non-pharmacologic interventions applicable in primary care settings. The synthesized findings were then presented narratively.

Musculoskeletal manifestations of diabetes mellitus

This review explores the diverse range of musculoskeletal complications associated with DM, which can significantly impair mobility and quality of life. The conditions discussed include diabetic cheiroarthropathy, characterized by stiffness and thickened skin of the hands; adhesive capsulitis, involving painful restriction of shoulder movement; and DISH, marked by abnormal calcification along the spine. Additionally, we examine CTS and trigger finger, both of which result from nerve compression and tendon thickening, respectively. Finally, the review covers Charcot joint, a serious complication of diabetic neuropathy leading to joint destruction.

Diabetic cheiroarthropathy (limited joint mobility)

Diabetic cheiroarthropathy, also known as diabetic stiff hand syndrome, is characterized by thickened, waxy skin on the dorsum of the hands and progressive limitation in the range of motion, especially of the small joints of the fingers [2,12].

Clinical Features, Pathophysiology, Relevance to Diabetes and Management

Diabetic cheiroarthropathy is characterized by symmetrical stiffness and flexion contractures of the fingers. Patients are often unable to fully extend their fingers, with the fourth and fifth digits most commonly affected. A positive “Prayer Sign” or “Tabletop Sign,” in which patients are unable to approximate their palms or lay their hands flat on a table, is a typical finding [12,13]. The underlying mechanism involves chronic hyperglycemia, which promotes the formation of AGEs. These AGEs accumulate in the skin and periarticular connective tissues, leading to collagen cross-linking, reduced tissue elasticity, and thickening of tendons and ligaments [6,7]. Diabetic cheiroarthropathy occurs in up to 30% of patients with long-standing type 1 or type 2 diabetes and is strongly associated with microvascular complications such as retinopathy and nephropathy [12,13]. Management primarily focuses on optimizing glycemic control, along with supportive interventions such as stretching exercises, physical therapy, and pain management using topical or systemic analgesics [12,13].

Adhesive capsulitis (frozen shoulder)

Adhesive capsulitis, commonly referred to as frozen shoulder, involves inflammation and fibrosis of the glenohumeral joint capsule, leading to pain, stiffness and progressive loss of shoulder motion [2,12,14-17].

Clinical Features, Pathophysiology, Relevance to Diabetes and Management

Frozen shoulder, or adhesive capsulitis, typically presents with a gradual onset of shoulder pain that is followed by progressive stiffness. Patients experience a loss of both active and passive range of motion. In individuals with diabetes, the condition is often bilateral, although it may initially present unilaterally [12-17]. The pathophysiology involves chronic hyperglycemia, which induces collagen abnormalities and fibroblast proliferation, leading to thickening and contracture of the joint capsule. In addition, inflammation of the synovial tissue contributes to pain and worsening stiffness [6,7,14-17].

The prevalence of frozen shoulder in the general population is estimated to be 2-5%. However, patients with diabetes are at substantially higher risk, with reported prevalence ranging from 10% to 20%. Compared to non-diabetic individuals, diabetic patients also demonstrate a markedly higher rate of bilateral involvement, with estimates of 33-42% compared to 5-20% in non-diabetic subjects [18].

Management depends on the severity of symptoms. Mild cases typically respond well to conservative measures, including analgesics and physiotherapy, with intra-articular corticosteroid injections required only in selected patients. Symptoms may persist for more than one to two years in fewer than one-third of cases, and these patients may benefit from interventions such as capsular hydrodistension [16,17]. Nonsteroidal anti-inflammatory drugs (NSAIDs) or corticosteroid injections used judiciously in diabetic patients can provide symptomatic relief. Physical therapy remains the cornerstone of management, aimed at preserving and restoring range of motion. In refractory cases, surgical options such as capsular release may be necessary [12-15].

Diffuse idiopathic skeletal hyperostosis

DISH is a condition characterized by abnormal calcification and ossification of ligaments and entheses, particularly along the anterior longitudinal ligament of the spine [2,12,17,18].

Clinical Features, Pathophysiology, Relevance to Diabetes and Management

DISH is characterized by stiffness and reduced spinal mobility, most prominently affecting the thoracic and lumbar spine. The condition may be asymptomatic or may present with chronic back pain. When the cervical spine is involved, patients can develop dysphagia due to mechanical compression [12,13,17,18]. The underlying pathophysiology is thought to involve insulin and insulin-like growth factors, which stimulate osteoblastic activity and promote abnormal bone formation. Hyperinsulinemia and obesity are also recognized as significant risk factors [6,7]. DISH is more common in patients with type 2 diabetes, with studies suggesting a prevalence as high as 13% in diabetic populations. Management focuses primarily on symptomatic relief with analgesics and physical therapy. Regular exercise is recommended to help maintain spinal mobility, while addressing metabolic risk factors such as obesity and hyperglycemia is essential in reducing disease progression [12,13,17,18].

Carpal tunnel syndrome (CTS)

CTS is caused by compression of the median nerve as it passes through the carpal tunnel in the wrist, resulting in sensory and motor disturbances [2,12].

Clinical Features, Pathophysiology, Relevance to Diabetes and Management

CTS in patients with diabetes commonly presents with numbness, tingling, and a burning sensation in the thumb, index finger, middle finger, and the radial half of the ring finger. Symptoms frequently worsen at night, and in advanced cases, patients may develop thenar muscle atrophy and weakness. Clinical examination often reveals a positive Tinel’s sign and Phalen’s test [12,13]. The underlying pathophysiology involves glycation of connective tissue structures, particularly the transverse carpal ligament, along with contributions from diabetic neuropathy and tissue edema, which together promote compression of the median nerve [6,7].

The prevalence of CTS among individuals with diabetes ranges from 14% to 30%, which is significantly higher than in the general population. Long-standing diabetes and poor glycemic control further increase the risk, and women are affected more commonly than men [18]. Initial management consists of nocturnal wrist splinting for a minimum of three weeks. If symptoms persist, treatment options include corticosteroid injections, NSAIDs, and vitamin B6 supplementation, all of which have been shown to reduce inflammation and swelling. Steroid injections are performed more frequently in diabetic patients, with estimates suggesting a rate 4-14 times higher than that of the general population, and they achieve a high success rate of nearly 90%. For patients who fail conservative treatment, surgical decompression remains the definitive option [17-19].

Trigger finger (stenosing tenosynovitis)

This is a condition where inflammation and thickening of the flexor tendon sheath cause the finger to catch or lock during flexion [2,12].

Clinical Features, Pathophysiology, Relevance to Diabetes and Management

Trigger finger is characterized by painful clicking, locking, or snapping during finger movement. Patients often experience stiffness, particularly in the morning, and nodularity can be palpated over the flexor tendon at the base of the affected digit. In some cases, passive manipulation is required to fully extend the digit [12,13]. The underlying pathology involves chronic inflammation and fibrosis within the tendon sheath, which impair smooth tendon gliding. In addition, hyperglycemia-induced glycation contributes to reduced tendon elasticity [6,7,17,18]. Prevalence of flexor tenosynovitis is approximately 1% in the non-diabetic population but rises to 10-20% in individuals with diabetes. The likelihood of developing this condition increases substantially with the duration of diabetes [18]. Management depends on disease severity: mild cases may be treated with splinting and NSAIDs, while refractory or severe cases may require corticosteroid injections into the tendon sheath or, in persistent cases, surgical release [12,13,17,18].

Charcot joint (neuropathic arthropathy)

Charcot joint is a progressive, destructive joint disease resulting from neuropathy, leading to joint dislocation, deformity, and instability, most commonly affecting the foot and ankle [2,12,17,18].

Clinical Features, Pathophysiology, Relevance to Diabetes and Management

Charcot arthropathy typically presents with a red, swollen, and warm joint, which is often misdiagnosed as cellulitis or gout. Because of decreased pain perception due to underlying neuropathy, patients may not recognize the severity of the condition. Progressive foot deformities, such as a rocker-bottom foot, can develop, significantly increasing the risk of ulceration and secondary infection [12,13,17,18,20,21]. The underlying mechanism involves peripheral neuropathy, which leads to a loss of protective sensation. Repetitive trauma, combined with impaired microvascular function, results in bone resorption, joint collapse, and deformity [6,7,17,18].

Patients with longstanding diabetes, with an average duration of approximately 15 years, are at a greater risk of developing peripheral neuropathy and subsequently Charcot arthropathy. The condition affects nearly one in 700 diabetic patients [18]. Management centers on avoidance of weight-bearing, most often through casting, which remains the mainstay of treatment and should be continued for at least three months or until erythema and swelling have resolved. Additional strategies include close monitoring for radiographic improvement, use of NSAIDs, and administration of calcitonin. Surgical reconstruction is reserved for severe or unstable cases. Patient education is crucial to reduce the risk of recurrence and prevent foot ulceration [12,13,17,18,20].

A summary of the most common musculoskeletal conditions observed in patients with diabetes is provided in Table 1.

**Table 1:** Summary of Major Musculoskeletal Disorders Associated with Diabetes Mellitus This table summarizes the clinical features, pathophysiological mechanisms, prevalence among diabetic populations, and primary management strategies for musculoskeletal conditions commonly associated with diabetes mellitus. Conditions include diabetic cheiroarthropathy, adhesive capsulitis, DISH, CTS, trigger finger, and Charcot joint. These disorders contribute significantly to disability and functional decline in diabetic patients and should be recognized in routine primary care. AGEs: advanced glycation end-products ~: nearly PT: physical therapy ROM: range of motion NSAIDs: non steroidal anti inflammatory Drugs DISH: diffuse idiopathic skeletal hyperostosis T2DM: type 2 diabetes mellitus CTS: carpal tunnel syndrome References: [2,6,7,12,13-21]

Condition	Clinical Features	Pathophysiology	Relevance to Diabetes	Management
Diabetic cheiroarthropathy	Thickened skin, flexion contractures, and limited finger mobility	AGE accumulation leads to stiffening of connective tissue and tendon thickening	Seen in ~30% of diabetics	Glycemic control, stretching, PT and analgesics
Adhesive capsulitis	Progressive shoulder pain, bilateral stiffness, and limited ROM	Fibrosis and thickening of glenohumeral joint capsule	10–30% prevalence in diabetics; more likely bilateral	NSAIDs, corticosteroid injections, PT, and surgery in severe cases
DISH	Stiff spine, back pain, and possible dysphagia if cervical spine is involved	Calcification/ossification of spinal ligaments	More common in T2DM; up to 13% prevalence	PT, pain management, and lifestyle modifications
CTS	Numbness and tingling in hand, and nocturnal symptoms	Median nerve compression due to connective tissue glycation and swelling	14–30% prevalence; associated with long-standing diabetes	Splinting, NSAIDs, corticosteroid injections, and surgical decompression if severe
Trigger finger	Painful finger locking, clicking/snapping sensation, and morning stiffness	Tendon sheath thickening due to chronic inflammation and glycation	10–20% prevalence in diabetes; often affects multiple fingers	Splinting, steroid injections, and surgical release
Charcot joint	Warm, swollen joint, and rocker-bottom foot deformity	Neuropathy leads to joint destruction	Rare (<1%) but serious; often in foot/ankle with diabetic neuropathy	Urgent specialist referral, casting, foot care education, surgery

Pathophysiology and risk factors

Hyperglycemia leads to the formation of AGEs, which accumulate in collagen and other connective tissues, causing stiffness and reduced elasticity. Chronic inflammation further promotes fibrotic changes. Diabetic microvascular complications, including neuropathy and retinopathy, impair joint proprioception and healing. Risk is worsened by sedentary behavior, poor glycemic control, and obesity [6,7].

Diagnostic approach in primary care

PCPs should suspect diabetes related MSK complications in patients presenting with unexplained stiffness, joint pain, or limited range of motion. This is of utmost importance when symptoms are symmetric or bilateral. Physical examination supported by imaging remains essential. Imaging such as X-rays or magnetic resonance imaging (MRI) can be considered in conditions like DISH or Charcot joint. Red flag signs-such as acute joint swelling, erythema, or systemic symptoms-warrant immediate referral to specialists [2,8-11].

Management strategies

Lifestyle Interventions

Physical activity: Regular exercise is foundational to managing both diabetes and associated MSK conditions. It improves insulin sensitivity, enhances joint mobility, and reduces systemic inflammation. Low-impact aerobic activities such as cycling, walking, or stationary biking, and practices like yoga or tai chi, play a crucial role in managing MSK complications in diabetes. Walking helps improve circulation and supports functional independence. Cycling promotes joint mobility with minimal mechanical stress. Yoga and tai chi contribute to improved balance, flexibility, and proprioception. Additionally, integrating light resistance training is essential for muscle strengthening. It stabilizes joints and helps reduce pain associated with mechanical overload [12-17,18,20].

Weight reduction: Reducing body weight by even 5-10% significantly decreases biomechanical stress on joints (particularly knees, hips, and spine), while also improving glycemic control. Weight loss helps reduce adipokine-driven inflammation, which contributes to both insulin resistance and degenerative joint processes [12-17,18,20].

Glycemic control: Tight blood glucose control reduces the formation of AGEs, which stiffen tendons and periarticular tissues. Improved glycemic status also slows progression of diabetic neuropathy, thus reducing the risk of Charcot joint development and related deformities [20,22].

Pharmacological Management

Analgesics and anti-inflammatory agents: For mild to moderate MSK pain, acetaminophen is often recommended as a first-line agent due to its favorable safety profile. Topical NSAIDs, such as diclofenac gel, offer targeted relief for localized joint or tendon inflammation with minimal systemic absorption, making them particularly suitable for patients with renal impairment or high cardiovascular risk. While systemic NSAIDs can be effective and may be appropriate for younger or low-risk individuals, their use requires caution due to the potential for nephrotoxicity, gastrointestinal bleeding, and cardiovascular complications [12-17,18,20].

Neuropathic pain management: Conditions such as Charcot joint and diabetic neuropathy carry a neuropathic component to them. Pharmacologic management includes agents that target nerve-related pain. Medications such as pregabalin and gabapentin are effective for modulating neuropathic pain but may require renal dose adjustments due to their primary renal excretion. Tricyclic antidepressants (TCAs), such as amitriptyline, when taken at low doses, can be considered for their analgesic properties. Serotonin-norepinephrine reuptake inhibitors (SNRIs), including duloxetine, offer a dual benefit. These address both musculoskeletal pain and diabetic neuropathy, making them a valuable option in patients with overlapping pain syndromes [18,20,22,23].

Corticosteroids: Intra-articular steroid injections may be used for severe trigger finger or adhesive capsulitis. However, repeated injections should be avoided due to risk of tendon rupture [18].

Physical Therapy and Rehabilitation

Interventions: Rehabilitation intervention strategies constitute a combination of active and passive range of movement (ROM) exercises to prevent stiffness and maintain joint mobility. Manual therapy techniques, such as joint mobilizations and soft tissue manipulation, are used to enhance flexibility in tendons and fascia. Stretching protocols are essential for addressing issues like trigger finger or joint rigidity. Functional retraining focuses on teaching adaptive techniques for daily activities such as dressing, reaching, or walking, thereby promoting independence. Additionally, assistive devices, such as bracing, splints, or orthotics, are recommended to support deformities associated with Charcot foot, and can improve stability and mobility [24-27].

Multimodal rehab plans:* *Incorporating pain management techniques such as transcutaneous electrical nerve stimulation (TENS), encouraging home exercise programs with clearer goals and scheduled follow-ups, to assess progress and compliance [27,28].

Management of diabetes related MSK conditions requires a multifactorial approach. This includes lifestyle modification, pharmacologic treatment, and rehabilitation, as outlined in Table 2.

**Table 2:** Management Strategies for Musculoskeletal Complications in Diabetes Mellitus This table outlines evidence-based approaches to managing musculoskeletal conditions commonly seen in diabetic patients. Strategies are grouped into three key domains: lifestyle interventions, pharmacological management, intervention and physical therapy and rehabilitation. Each category includes specific interventions designed to improve joint function, reduce pain, and address underlying metabolic contributors such as inflammation and poor glycemic control. These multidisciplinary approaches aim to prevent disability, enhance mobility, and improve overall quality of life in diabetic individuals. AGEs: advanced glycation end-products NSAIDs: non steroidal anti-inflammatory drugs ROM: range of motion TENS: transcutaneous electrical nerve stimulation References: [12-17,18,20,22-28]

Domain	Strategy	Details & Examples
Lifestyle Interventions	Physical activity	Low-impact exercises; walking, aquatic therapy, yoga, tai chi; resistance training to support joint stability
	Weight reduction	Target 5–10% weight loss reduces mechanical joint stress and systemic inflammation
	Glycemic control	Slows AGE formation-Improves insulin sensitivity and pain perception
Pharmacological Management	Analgesics	First-line: acetaminophen; Topical NSAIDs for localized pain; Systemic NSAIDs used cautiously in low-risk patients
	Neuropathic pain agents	Gabapentin, pregabalin for nerve-related pain; duloxetine or amitriptyline for dual benefit
	Corticosteroid injections	For adhesive capsulitis or trigger finger
Physical Therapy & Rehabilitation	Interventions	Includes passive and active ROM exercises to maintain mobility and reduce stiffness (e.g., adhesive capsulitis, cheiroarthropathy); manual therapy and stretching for fascia/tendon flexibility and contracture prevention; strength and balance training for neuropathy; bracing/splints (e.g., Charcot foot)
	Multimodal rehabilitation plans	Use of TENS; structured home programs with periodic follow-up to monitor adherence and progress

Multidisciplinary integrated care and challenges in primary care

PCPs should collaborate with specialists, including endocrinologists, ophthalmologists, rheumatologists, and pain specialists for complex or refractory cases. Additionally, diabetes educators should be involved to reinforce patient self-management and adherence [29]. PCPs often face barriers, such as underrecognition of MSK pain as a diabetic complication and time constraints. Moreover, lack of clear guidelines addressing the intersection of MSK and diabetic care limits proactive screening and standardization in care [30,31].

## Conclusions

MSK complications are a significant yet underrecognized aspect of DM. They contribute to pain, disability, and reduced quality of life. Common conditions such as diabetic cheiroarthropathy, carpal tunnel syndrome, adhesive capsulitis, DISH, trigger finger, and Charcot arthropathy frequently affect individuals with poorly controlled or long-standing diabetes. Although highly prevalent, these disorders are often overlooked in routine care. PCPs, who manage most diabetes cases, are well-positioned to identify MSK complications early and initiate appropriate interventions. Through thorough physical assessments, timely referrals, and implementation of evidence-based strategies (weight management, glycemic control, exercise, pharmacological treatments, and physical therapy), PCPs can help prevent progression and preserve function. Multidisciplinary collaboration with diabetes educators and specialists, further enhance outcomes. In conclusion, MSK screening and treatment may be integrated into diabetes management protocols, supported by clearer guidelines, improved awareness, and system-level support. Addressing these complications, empowers providers to deliver more comprehensive, holistic, and patient-centered diabetes care
